# Management of Open Pediatric Fractures: Proposal of a New Multidisciplinary Algorithm

**DOI:** 10.3390/jcm12196378

**Published:** 2023-10-06

**Authors:** Angelo Gabriele Aulisa, Martina Marsiolo, Luca Basiglini, Cristian Aletto, Marco Giordano, Francesco Falciglia

**Affiliations:** 1 U.O.C. of Orthopaedics and Traumatology, Bambino Gesù Children’s Hospital, IRCCS, 00165 Rome, Italy; agabriele.aulisa@opbg.net (A.G.A.); luca.basiglini@opbg.net (L.B.); marco.giordano@opbg.net (M.G.); francesco.falciglia@opbg.net (F.F.); 2 Department of Human Sciences, Society and Health, University of Cassino and Southern Lazio, 03043 Cassino, Italy; 3 Department of Musculoskeletal Disorders, Faculty of Medicine and Surgery, University of Salerno, 84084 Baronissi, Italy; cris.aletto28@gmail.com

**Keywords:** open pediatric fracture, management of open fractures, multidisciplinary algorithm, exposed fractures, children’s fractures

## Abstract

Background: An algorithm for managing open fractures in children is still being debated; the present study suggests an evidence-based way to manage these patients in the emergency department. Methods: The literature on “Open fractures in children” was carefully analyzed using keywords. The primary sources were The Cochrane Library, PubMed, and Researchgate. Conclusion: We proposed an evidence-based algorithm for managing open fractures in children to standardize clinical practice and improve the care of these patients.

## 1. Introduction

Orthopedic surgeons have long debated the acute management of open fractures, especially in children, because incomplete or inappropriate treatment could cause severe complications in patients. Open fractures constitute between 0.7% and 2% of all pediatric fractures, and they must be considered orthopedic emergencies due to the high risk of contamination, osteomyelitis, malformation, nonunion, and disability [1]. Demographics and injury mechanisms vary widely; most studies report a higher incidence in male patients due to high-energy injuries (falls from heights and motor vehicle accidents). The most common sites of injury reported are the fibula or tibia (35%); the second most frequent site is the forearm (32%), followed by the hand or metacarpals bone (10%); and the least frequent sites are the feet (5%) and the elbow 2% [2], with an incidence between 1% and 9% of all pediatric fractures [3].

There are several differences in the bone physiology of children and adults. In children, the thick periosteum limits the displacement of fractures; it is also more active and vascularized, which permits rapid fracture healing in young children compared with older children and adults. Children generally regenerate bone more quickly in the face of bone loss. Moreover, they have a minor risk of infection with open fractures of the upper and lower extremities compared with adults; however, they have a higher risk of growth disturbances. Moreover, they have a large surface area compared to body mass, so they are more prone to hypothermia; at the same time, they have an excellent physiological reserve, maintaining blood pressure until at least 25% of blood volume is lost [4]. Because of these differences, emergency physicians need to develop an algorithm focused on open pediatric fractures to manage them better. 

## 2. Materials and Methods

We performed a literature review according to the Preferred Reporting Items for Systematic Reviews and Meta-Analysis (PRISMA) guidelines, considering articles published from 2000 up to July 2023. A literature search identifying all articles involving the management of open fractures in children was performed by consulting PubMed. Keywords included the terms “children” AND “open fracture,” “management AND open fracture,” “Algorithm AND open fracture AND children”, and “pediatric AND open fracture”, and they were differently combined. After removing duplicates and identifying the relevant studies through abstract information, the full text was examined, applying pre-established inclusion and exclusion criteria. The inclusion criteria were review studies, peer-reviewed studies, articles written in English, comparative studies, controlled clinical trials, observational studies, and randomized clinical trials based on the management of pediatric open fractures. Case reports, conference presentations, narrative reviews, letters to the editors, editorials, and expert opinions were excluded. The subsequently applied exclusion criteria were articles that did not focus on the management of open fractures, articles that focused on less frequented breaking sites, and reports that did not focus on humans. The authors prioritized articles that included information on algorithms and management, as well as the latest articles. The authors decided on article inclusion by consensus. Priority was placed on meta-analyses, randomized controlled trials, and systematic reviews. All data were collected in a datasheet and included the study authors, publication year, type of article, and what management area was paid attention to.

## 3. Results

**Figure 1:**
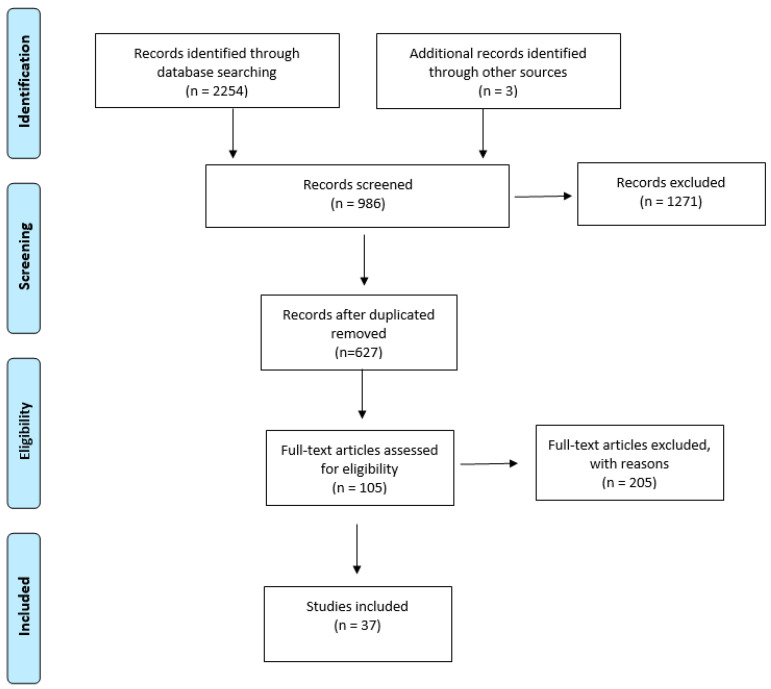
Prisma flowchart.

A total of 2257 articles were identified after searching the databases; they were analyzed based on titles. After excluding studies on open fractures in adult patients and studies focused on small segments, 986 articles were obtained. After excluding duplicates, 627 studies were analyzed based on their abstracts. After this screening, 310 articles were selected, and among them, only 105 articles were analyzed based on their full text. After applying the mentioned exclusion criteria, 37 studies were finally used for the present article (Figure 1).

### 3.1. First Evaluation

The initial assessment of children with open fractures begins with a primary survey, assessing the patient’s condition rapidly and accurately following the PALS (pediatric advanced life support) and ATLS (advanced trauma life support) guidelines [3]. Orthopedic management should focus on saving the limb: pulses, peripheral capillary refill, limb color, temperature, and bleeding from wounds indicate the vascular condition. The gross motor function and sensation of the limb should also be documented. In cases of pediatric combined vascular and orthopedic trauma, time is tissue, and prompt treatment is needed at the nearest level I or II trauma center. When transport to a level I trauma center is prevented by weather or distance, a level II trauma center is a critical intermediary providing resuscitation and damage control surgery. Pediatric patients with high-velocity GSWs present additional resuscitative challenges to emergency physicians. Their injuries are large relative to their limb size, and pediatric patients have a lower circulating blood volume than adults. Once resuscitated, vascular repair takes precedence over orthopedic treatment [5]. 

Compartment syndrome should be suspected in high-velocity limb trauma of the leg or forearm and assessed periodically. It is essential to consider that children tend to develop swelling and compartment syndrome later than adults and that the conventional 5Ps (pain, pallor, paresthesia, paralysis, and pulselessness) are not always reliable. The 3As—increasing analgesic requirement, anxiety, and agitation—are generally more helpful to assess compartment syndrome in children [6]. Intra-compartment pressure is not widely used in pediatric practice; when there is a severe clinical suspicion, open fasciotomy of all affected compartments should be urgently carried out, and wounds should be left open despite the increasing chances of infection [7].

In the emergency room, details of all open wounds should be recorded. A photographic record of the open wound should be made to avoid repeated dressing disturbances, which increase the risk of infection [8]. The wound must be classified according to a modified post-debridement Gustilo and Anderson classification because the standard Gustilo classification seems unclear and does not reflect the extent of soft tissue and skeletal damage. Factors such as the degree of soft tissue damage and periosteal stripping are more critical than the wound size in children [9] (Table 1). However, the Gustilo and Anderson (GA) classification has a limited inter-observer reliability, with concordance as low as 60%, and the authors built the classification on adult patients [10]. 

In children’s injuries, it is more important to pay attention to how much damage there is to the soft tissue and the stripping of the bone covering rather than just the size of the wound. Unlike in adult open fractures, pieces of bone that may not be healthy should be kept, as they become a part of the healing process for the fracture [11]. 

### 3.2. Antibiotic Therapy

When treating open fractures, one crucial aim is to prevent bone and soft tissue infection. It has been found that, for every minute of delay in going to the hospital, the chances of infection increase by 1% [12]. In type 1 GA, the infection rate is about 2%, and systemic antibiotic delivery to the zone of injury is more effective. Soft tissue devascularization and necrosis associated with types 2 and 3 GA promote bacterial colonization and growth within the area of injury and limit the accessibility of systemic antibiotic therapy. Infection rates range from 2% to 15% for type 2 fractures and from 5% to 50% for type 3 fractures. Prompt antibiotic administration is a crucial way to minimize the risk of infection associated with open fractures. In the literature, there is consensus about using cephalosporin alone for type I and type II open fractures, with an aminoglycoside added for type III fractures. Treatment typically lasts for 48 h or less. A delay of 6 h seems to be accepted for type I and type II fractures but not for type III fractures [13,14]. A first-generation cephalosporin (Cefazolin 100 mg/kg/day) divided into doses given every eight hours (up to a maximum of 2 g every eight hours) is typically administered to patients with an open fracture. Patients with type II or III open fractures are additionally given an aminoglycoside (gentamicin 5 to 7.5 mg/kg/day) to enhance Gram-negative coverage divided into doses every eight hours. The East guidelines support this association. Penicillin (150,000 units/kg/day) or one of its derivatives is added to cover anaerobes and Clostridium species when dealing with dirty wounds exposed to soil or vascular injuries. We can suggest that, when used prophylactically, antibiotic administration should not exceed 72 h; by this time, micro-biological studies will be available to guide appropriate antibiotic choice [15].

### 3.3. Cultures

Primary wound cultures were routinely practiced because prophylactic antibiotic administration and aggressive operative debridement were not performed. Today, the empiric treatment of long-bone fractures consists of immediate prophylactic antibiotics, irrigation, and debridement; in fact, it is widely demonstrated that there is no benefit in initial wound cultures [16]. The majority of studies indicate that the initial flora present in open fracture wounds are not responsible for the infection, and pre-debridement wound cultures are not helpful in predicting post-debridement wound infection; moreover, in most cases, infection of the wound occurs due to hospitalization [17,18,19].

### 3.4. Secondary Survey 

During the second survey, the medical staff should check whether the patient has received a tetanus shot. If not, they should give the patient a tetanus vaccine. Orthopedic doctors have to check the wound; if it is clean, they must consider whether the patient has had a tetanus shot in the last ten years. If they have not had one, or if it is not known, they should give them a vaccine [3,20] (Table 2 and Table 3).

### 3.5. Trauma Team: Management in the Emergency Department

Once any emergency procedures are completed and the patient is stabilized, the trauma team is activated, which includes the emergency doctor, the resuscitation anesthesiologist, the general surgeon, the plastic surgeon or orthoclastic surgeon, and the orthopedic surgeon; the recent literature shows that even the presence of a psychologist can be helpful for management in the case of wide soft tissue loss to better manage patient care [21]. Pediatric patients with high-velocity GSWs present additional resuscitative challenges to emergency physicians. Their injuries are large relative to their limb size, and pediatric patients have a lower circulating blood volume than adults. Once resuscitated, vascular repair takes precedence over definitive orthopedic fixation. This is especially important to reduce ischemia and reperfusion injury in the setting of transferred patients with an increased injury-to-surgery time. These patients should have temporizing bony fixation and revascularization preferably with native vessel repair, or a reverse saphenous graft if native repair is not feasible. The surgical sequence of temporizing orthopedic external fixation to allow revascularization should be performed as a bridge to future definitive internal fixation in congruence with damage control orthopedics [5,22].

After life-saving procedures, we focus primarily on bleeding control and wound care. Direct pressure should be applied to control bleeding, and, if possible, the affected limb should be elevated. Immediate wound irrigation should be performed using a sterile saline or soapy solution to reduce the risk, and prophylactic antibiotic therapy should be started based on evidence. Once the patient is stable, imaging studies, such as X-rays, computed tomography (CT), or ultrasound scans, should be performed. In the presence of frank skin or soft tissue lesions and with the clinical or instrumental suspicion of vascular lesions, the trauma team should be assisted by the plastic surgeon and the vascular surgeon. If compartments are tense or have disproportionate pain with a passive stretch by the fingers, compartment syndrome should be suspected, and compartment pressures should be measured [2]. Orthopedic surgical management and timing depend on several factors, including the child’s stability and the severity of the fracture, such as the modified Gustilo–Anderson classification. There is consensus in the literature on treating pediatric patients with a type I Gustilo–Anderson fracture with primary irrigation. In contrast, type II and III fractures often require a procrastinated wound closure and may require a skin graft or flap [23]. After grading the lesion, extensive or contaminated wounds should be irrigated with copious volumes of saline solution. Small foreign bodies should be removed from the wound with a sterile technique. The debridement and irrigation of exposed fractures in the emergency room are some of the most critical stages in treating exposed fractures in children. The initial debridement in children should be more conservative than that in similar wounds in adults. In the pediatric population, fracture fragments with questionable viability and soft tissue attachment should be preserved because they may promote fracture healing [24]. Although surgical site infection is one of the most common and serious complications of orthopedic trauma surgery in adults, in children, the rates are lower, and when it occurs, the prognosis of children is superior to that of adults. Early infection is more common in adult patients, which indicates that children have a superior capacity for soft tissue recovery [25,26,27,28].

While there are guidelines for the irrigation volume of exposed fractures in the adult population, there are few recommendations for the pediatric population. This is because excessive irrigation can extravasate in soft tissues, increasing the risk of compartment syndrome and delaying or even preventing bone healing. The volume of irrigation should be chosen considering the size of the wound, the size of the patient, and the contamination rate [29]. The order of debridement is summarized as follows:

- The excision of necrotic tissue from the margins of the wound.
- The extension of the wound to adequately explore the fracture ends.
- The debridement of the wound margins to the bleeding tissue.
- The resection of the skin, adipose tissue, muscle tissue, and fascia contaminated in necrosis.
- Fasciotomies, if necessary.
- The meticulous irrigation of fracture and wound heads.

### 3.6. Choice of Treatment: When and Which

Fracture stabilization is essential to reduce pain, prevent further damage, decrease the inflammatory response, and promote early mobilization. Damage control is also fundamental in managing polytraumatized patients with severe health conditions and patients who need immediate stability to manage vital parameters and facilitate patient care in intensive care units. Moreover, this subgroup of patients, the most complex to treat due to the probable multiple comorbidities, should be converted to definitive internal or external fixation as soon as possible. However, in stable patients, the treatment should be chosen considering several variables, such as the fracture’s displacement, the patient’s age, and the degree of exposure, according to Gustilo–Anderson. In type I Gustilo–Anderson fractures, intravenous antibiotic therapy associated with exposure lavage is sufficient to prevent infections, whether treated conservatively or surgically, and the management of the wound can be carried out in the emergency room [30,31,32]. Gustilo II and III A open fractures can be treated using debridement and internal fixation, given the low risk of infection. External fixation in pediatric patients has shown more complications than in adult patients, such as pin tract infection and a longer healing time; whenever possible, the use of IM devices for the treatment of open femur fractures in children should be considered, especially for grade I open injuries. If EFs are used, avoiding malunion may decrease the refracture rate, and the primary application of, or secondary change to, an IM device can be considered [33,34]. Time to surgery did not vary by fracture grade, although there was a trend toward more rapid surgical interventions in the grade 2 and grade 3 injuries than in the grade 1 fractures; in the literature, it was reported that, despite a delay in surgery of 6 h, there was no difference in the overall rate of infection [35,36].

### 3.7. Use of Vac Therapy

Using VAC therapy to treat soft tissue defects related to wounds has been helpful for orthopedists. It helps them cover and close the wounds more effectively. The use of VAC has been postulated to decrease edema and purulent drainage (via the removal of bacteria) and increase blood flow, thereby promoting granulation tissue, cell/protein synthesis, and healing [37]. Moreover, the use of VAC seems to be safe and effective in reducing the number of infections in children with open fractures. It is important to see this as a helpful tool for doctors who treat these types of injuries. Also, since it is easy to change dressings, patients can move more easily, and the wound is closed off and sealed; using wound VAC not only reduces infection but is also helpful for children because it is easy to use [38].

### 3.8. Post-Operative Rehabilitation

As of now, open fractures in children are caused in most cases by street mischances and, most often, influence the leg. They are often combined with different skin and soft tissue defects; this is because the ability of young children to escape from risk on their own is poor. Due to the presence of wounds, they have trouble in functional recuperation, unlike in closed fracture cases. Achieving functional recovery in a short time is imperative since, otherwise, the hospitalization time is extended.

Although the recuperating and remodeling capacity of young children is powerful, poor results can still be obtained due to insufficient treatment and restoration and children’s non-cooperation. In recent years, an unused concept in surgery has been created in Europe and America, named Upgraded Recuperation After Surgery (Times); this has been utilized broadly in grown-ups [39]. In a later consideration, Paerhati Rexiti et al. used this convention in young children. They combined an arrangement of methods consisting of fast recovery, light-wave anti-inflammatory, and lymphatic reflux tools related to taping; this method permitted quick recovery and a decreased utilization of antimicrobials, and these results are in agreement with those of other authors who illustrated a faster recovery in patients treated with the Times convention. Bright radiation can kill all sorts of organisms, including bacterial propagules, spores, Mycobacterium, infections, organisms, Rickettsia, and mycoplasma; this treatment, related to lymphatic massage, taping (which could reduce swelling), and early mobilization with the assistance of a physiotherapist, permits a quick recovery and requires less relief treatment (antimicrobial and anti-inflammatory treatments) [1].

## 4. Discussion

The assessment must be performed by a multidisciplinary team, and the first specialist must be the resuscitation anesthesiologist. The initial assessment of children with open fractures begins with a primary survey, assessing the patient’s condition rapidly and accurately following the PALS (pediatric advanced life support) and ATLS (advanced trauma life support) guidelines [3]. Subsequently, all the other members of the trauma team must be involved, bearing in mind that there are several differences between children and adult patients affected by open fractures. 

In high-velocity limb trauma of the leg or forearm, it is important to suspect the presence of compartment syndrome; children tend to develop swelling and compartment syndrome later than adults. Erdos et al. analyzed the diagnostic procedure for compartment syndrome in 24 children from arrival at the hospital until discharge, and they reported difficulties in making a diagnosis given the patients’ inability to express themselves; they found that the 3As—increasing analgesic requirement, anxiety, and agitation—are generally more helpful in assessing compartment syndrome in children [6]. Moreover, the intra-compartment cannot be measured until appropriate anesthesia is administered, especially in children younger than 5 years, so when there is a severe clinical suspicion, open fasciotomy of all affected compartments should be urgently carried out, and wounds should be left open despite the increasing chances of infection. Flynn et al. demonstrated that good results can be achieved even when fasciotomy is performed in the acute swelling phase, often twenty-four to forty-eight hours after the initial injury in pediatric patients [7].

Long-bone fractures also differ in skeletally immature patients. Growth plates are unique to children, and injury to the ends of long bones adds another dimension of partial or complete growth arrest, not only requiring reconstructive surgery but also resulting in long-term morbidity. The thick periosteum makes closed reduction easy, as part of it remains intact in many fractures. Similarly, it helps fracture healing. It also limits the displacement of fractures, so the degree of displacement of a fracture is not always proportionate to the energy transmitted to the limb [4].

The evaluation of soft tissue lesions is essential, and it is important to make a correct classification of the skin lesion through exposure. The classification of Gustilo in children is less precise than in adults; in children, it is important to pay attention to the integrity of the periosteum. Faraj et al. conducted a study on 27 children with an open tibial fracture and a mean age of 10 years; they classified the wound with the Gustilo classification pre- and post-debridement, demonstrating that the Gustilo classification is not specific and does not reflect the extent of soft tissue and skeletal damage. The degree of soft tissue injury is not always related to the size of the wound. A very large wound caused by a sharp object has minimal associated soft tissue crush and, therefore, may carry a very good prognosis; moreover, the size of the wound in children is underestimated because of their relatively smaller body surface area compared to that of adults.

Factors such as the degree of soft tissue damage and periosteal stripping that are noticed following wound debridement and the velocity of injury are far more important than the wound size given the ability of the periosteum to regenerate bone and to keep the bone fragments in contact [9]. 

Once the type of exposure has been classified, it is necessary to decide regarding the need, the time, and the type of debridement. In the literature, it is reported that the debridement can be postponed to 24 h in GA I and II and within 6 h in III. In children, in selected cases, it can be delayed or may not be necessary (inside-out lesions), and soft tissue with doubtful viability does not need to be excised like isolated bone fragments. Moreover, excessive washing can increase the risk of compartmental syndrome and decrease bone healing [27]. 

Two recent studies have found that surgery may not be needed for all mild open fractures. Yang and Eisler discovered that none of their ninety-one patients, including thirteen children, who had a type I open fracture and did not receive surgery got any infections. In the only study we know of that looked at treating broken bones without surgery in only children, they found that one child out of forty got a serious infection [27,28].

Cultures of the wound are not necessary. Two recent articles and one review observed the bacteria isolated from exposed fracture wounds before and after infection, and they found that the initial flora are not the infecting organisms in open fracture wounds and that pre-debridement wound cultures have no value in predicting post-debridement wound infection [16,17,18].

Regarding the use of prophylactic antibiotic combination, it should be used only in lesions up to Gustilo type II and not for longer than 24–72 h [3,13,15]. Khun et al. investigated the risk factors for deep infection after an open long-bone fracture in 303 pediatric patients, and they found that the only factor that remained significant in multivariable regression was the duration between the injury and hospital presentation, where the odds of deep infection increased by 1% for every minute of delayed presentation [12]. 

Moreover, the risk of surgical site infection is low. In a recent study by Nandra at al., a review was performed on all open tibial fractures treated at a level-one pediatric trauma center between 2007 and 2015 (51 patients). The primary outcome was the rate of deep infection; they reported that, in children, the rate of deep infection is lower than that in adults, and when pediatric infections occur, they have a better prognosis than adult infections [26]. Chen et al. performed a retrospective study on 37 children with a mean age of 7.19 ± 2.28 years and 89 adults with a mean age of 40.38 ± 13.53 years who sustained open tibia fractures; they recorded data on age, gender, fracture site, Gustilo–Anderson grade, treatment management, and culture results from the infection. They reported that the surgical site infection rate of pediatric patients after open tibia fracture fixation is significantly lower than that of adults, and the prognosis of the former is superior. The results indicate a superior ability of soft tissue recovery and infection resistance after open tibia fracture fixation among children [25].

The choice of fixation in open pediatric fractures should fall on internal fixation whenever possible; recent studies have shown that this can be used as the first choice up to IIIA GA. External fixation (FE) in pediatric patients as a definitive treatment of open fractures involves more complications than internal fixation, a major risk of infection of the pin tract, a slower healing of the fracture, and an increased risk of refraction; internal fixation does not show an increased risk of osteomyelitis in treated patients compared to EF. Hong et al. conducted a study on 92 pediatric patients affected by open tibial shaft fracture; 55 patients (33 males and 22 females) were treated with EF, whereas 37 patients (21 males and 16 females) were included in the ESIN group. There was no statistically significant difference between the two groups concerning sex, age, body weight, duration from injury to surgery, Gustilo–Anderson classification, or concomitant injuries. There was no case of nonunion or malunion in either group. They found that angulation at the latest follow-up was higher in the EF group than in the ESIN group. The radiological union was faster in the ESIN group than in the EF group, and pin tract infection was the most troublesome complication in the EF group. Based on their study, ESIN is a viable option in selected patients of GA grades II and IIIA open tibial fractures with clinical outcomes comparable to those of external fixation [33]. Ramseirer et al. reported the treatment results of thirty-five patients affected by open femur fractures; EF was used in 23 cases, while 12 had an IM device (6 rigid nails and 6 elastic flexible nails). They recorded the complications, such as deep infections, pin track infections, refractures, varus malunions, valgus malunion, and leg length discrepancies exceeding 2 cm, and reported that treatment with IM devices had fewer complications than treatment with EF. They think that, whenever possible, the use of IM devices for the treatment of open femur fractures in children should be considered, especially for grade 1 open injuries [34]. 

## 5. Conclusions

**Figure 2:**
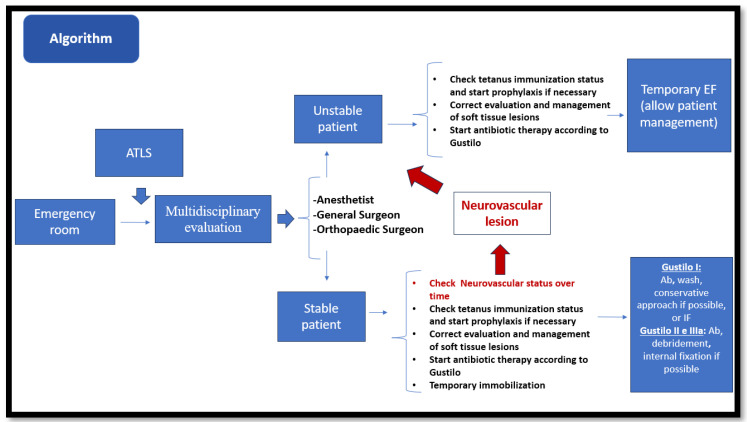
Proposal of an algorithm.

Based on the principles listed in these articles, we propose an algorithm for the management of open fractures in children. The authors believe that a standardized algorithm will improve care. A clear algorithm should be applied in every institution dealing with such trauma; ideally, a multidisciplinary approach should be the gold standard (Figure 2).

## Figures and Tables

**Table 1:** New classification of open fractures following the debridement of the wound.

Modified Gustilo and Anderson Classification
Type I A: wound requiring no skin graft or flap, level of contamination is minimal, the periosteum is intact with minimal to moderate muscle damage and no bone comminution
Type I B: wound requiring skin graft or flap, level of contamination is moderate to severe, the periosteum is intact with minimal to moderate muscle damage and no bone comminution
Type II A: wound requiring no skin graft or flap, level of contamination is minimal to moderate, the periosteum is stripped with minimal to moderate muscle damage and no bone comminution or minimal comminution
Type II B: wound requiring skin graft or flap, level of contamination is moderate to severe, the periosteum is stripped with minimal to moderate muscle damage, and bone comminution is minimal to severe or segmental fracture
Type III: wound requiring skin graft or flap, level of contamination is severe, the periosteum is stripped with muscular and neurovascular injury and unstable, comminute or segmental bone fractures with or without bone loss

**Table 2:** Recommendations about tetanus immunization.

Clean Wound with a Vaccine Dose within Ten Years	Not Reaccommodated
Wound > 1 cm deep, incurred > 6 h earlier, devitalized tissue, grossly contaminated → vaccine dose within 5 years	Not recommended
Wound > 1 cm deep, incurred > 6 h earlier, devitalized tissue, grossly contaminated → not received a tetanus immunization within the past 5 years or if their status is unknown	Recommended

**Table 3:** Immunization based on patient’s age.

Tetanus Immunization
Patient < 7 years old: DTaP (diphtheria, tetanus, and pertussis).
Patient between 7 and 10 years old: give Td (tetanus and diphtheria). For older children, give Tdap (tetanus, diphtheria, and pertussis).

## Data Availability

Datasets generated and/or analyzed during the current study are available from the corresponding author upon reasonable request.
